# Bridging the translational divide: identical cognitive touchscreen testing in mice and humans carrying mutations in a disease-relevant homologous gene

**DOI:** 10.1038/srep14613

**Published:** 2015-10-01

**Authors:** J. Nithianantharajah, A. G. McKechanie, T. J. Stewart, M. Johnstone, D. H. Blackwood, D. St Clair, S. G. N. Grant, T. J. Bussey, L. M. Saksida

**Affiliations:** 1 Genes to Cognition Programme, Centre for Clinical Brain Sciences, University of Edinburgh, Chancellors Building, 47 Little France Crescent, Edinburgh UK EH16 4SB; 2 Genes to Cognition Programme, The Wellcome Trust Sanger Institute, Hinxton Cambridgeshire UK CB10 1SA; 3 The Patrick Wild Centre, University of Edinburgh, Royal Edinburgh Hospital, Edinburgh UK EH10 5HF; 4 Division of Psychiatry, University of Edinburgh, Royal Edinburgh Hospital, Edinburgh UK EH10 5HF; 5 Institute of Medical Sciences, University of Aberdeen, Aberdeen UK AB25 2ZD; 6 Department of Psychology, University of Cambridge & The MRC and Wellcome Trust Behavioral and Clinical Neuroscience Institute, University of Cambridge, Cambridge UK CB2 3EB

## Abstract

Development of effective therapies for brain disorders has been hampered by a lack of translational cognitive testing methods. We present the first example of using the identical touchscreen-based cognitive test to assess mice and humans carrying disease-related genetic mutations. This new paradigm has significant implications for improving how we measure and model cognitive dysfunction in human disorders in animals, thus bridging the gap towards effective translation to the clinic.

There is a need for methods that address conserved mechanisms in rodents and humans, thus enabling translation. The last decade has seen increasing calls to develop improved, standardised assays for assessing behavior in animals, to not only advance robustness of scientific practice between laboratories, but the fundamental goal of effective medical translation. This need has been further fuelled by recent pharmaceutical industry cut-backs on central nervous system drug development due to a lack of effective outcomes from clinical trials. Rodent models of disease – with their financial and ethical advantages compared to non-human primates, and the relative ease of genetic and other manipulations – are an essential plank in this endeavor. However, one reason for the failures in clinical trials is likely the poor translational efficacy of the assays and measures employed to model clinical symptoms in rodents: pre-clinical testing of drugs for cognition is largely carried out using tests that have little in common with tests used in the clinical setting. A clear way forward in bridging the translational divide is the development of assays – ideally *identical* assays – that can be administered to both animals and humans, featuring objective and quantitative measures. Indeed, some progress to this end, mostly in non-human primate models, has already been made^1^,^2^,^3^,^4^. To this end, we present the first example of using the *identical touchscreen-based cognitive test* to assess complex problem solving in mice and humans carrying disease-related genetic mutations in homologous genes.

Testing cognition in humans and rodent models in an identical manner has traditionally been difficult, however we previously highlighted the development of the rodent touchscreen operant platform which has made this a possibility^5^. The rodent touchscreen tests use the same well-controlled, accurate and automated touchscreen methodology that is increasingly used in human testing (e.g., Cambridge Neuropsychological Test Automated Battery, CANTAB), providing a unique translational tool^6^. A recent study, which is the only example that comes close to this kind of cognitive translation, involved a mouse model with a mutation in a member of the postsynaptic Discs large homolog – Membrane Associated Guanylate Kinase (Dlg-MAGUK) family of scaffold proteins assessed on rodent touchscreen tests compared to human participants with copy number variations (CNVs) in the same gene assessed on 3 analogous – but *not* identical – CANTAB tests^7^.

While comparison of animals tested on rodent touchscreen tests to humans tested on human touchscreen tests is an improved approach to measuring similar underlying cognitive components, the likelihood of tapping into the same components would be maximised by employing *the same test in both species*. Therefore, to demonstrate the full translational capacity of the touchscreen assays, we employed the identical touchscreen test to assess both mice lacking the *Dlg2* gene (*Dlg2*^*−/−*^) and individuals with *DLG2* CNV deletions on the rodent version of the object-location paired-associates task. This test requires learning and remembering which of three objects (flower, plane, spider) is associated with one of three locations on the touchscreen (left, centre, right respectively) (Fig. 1a)^8^, therefore learning the paired-association between the shape and the object’s location. Control wild-type (WT) mice show a progressive increase in performance across blocks of training trials on this task, indicating intact visuo-spatial learning and memory. In comparison, *Dlg2*^*−/−*^mice show a robust impairment in object-location paired associates learning, with performance consistently around 50% (chance level) across training (Fig. 1b). Similar to WT mice, human control participants showed progressive acquisition of object-location paired associates across training trials, with no differences in performance due to either gender or IQ (Fig. 1c, Supplementary Fig. 1). Interestingly, however, individuals with *DLG2* CNV deletions tested on the same test failed to show this progressive acquisition and their performance was impaired compared to controls by the end of training, with performance still approximately at chance level (Fig. 1c, Supplementary Fig. 1). These results strikingly recapitulate the impairment that we observe in *Dlg2*^*−/−*^mice.

Human mutations in *DLG2* are rare but highly penetrant and have been reported in psychiatric disorders including schizophrenia^9^,^10^,^11^,^12^ and more recently autism^13^, intellectual disability^14^ and bipolar disorder^15^. Moreover, cognitive ability in the general population has also been shown to be influenced by genetic variation in the postsynaptic signalling complexes formed by MAGUKs, highlighting the importance of genes within these complexes – including *DLG2* – in regulating cognitive function^16^. This is evident within our data: of the 4 *DLG2* CNV carriers, one individual is clinically unaffected but showed a similar pattern of performance as the other 3 participants with clinical diagnoses in contrast to controls in this study and additionally, other CANTAB tests^7^. These findings highlight the need for future collaborative studies to increasingly identify individuals harbouring rare gene mutations and employ our approach of cognitive testing individuals based on their specific genetic architecture, both those with clinical diagnoses and unaffected individuals. In the current genomic era in which exome sequencing of large patient cohorts is routinely achievable, the genetic basis of neurological and neuropsychiatric disorders is rapidly being unravelled. Now more than ever, using animal models with targeted genetic manipulations combined with innovative behavioral methodology will be fundamental in effectively modelling disease-relevant cognitive dysfunction towards development of novel therapies. Our approach in developing identical assays that can be administered to both rodents and humans provides strong face validity which, although not guaranteeing construct or predictive validity without further work, is more likely to yield such ‘neurocognitive validity’ than the tasks that appear on the face of them to have little in common. This is not to say that more ecologically valid approaches are not desirable; indeed capitalising on species-specific behaviours can yield many advantages^17^. However for such approaches, just as with the touchscreen approach, the relevant criterion for utility in translation is neurocognitive validity. With the many advantages conferred by automated touchscreen testing^6^,^8^,^18^, the present study may represent the first example of what may become a new paradigm approach for effectively modelling cognitive dysfunction in animal models and effective translation to the clinic.

## Methods

### Animals

Homozygous knockout mice for *Dlg2* (*Dlg2*^−/−^) and WT littermates were generated from heterozygous intercrosses^19^ and maintained on a C57BL/6J background. Male mice (n = 10–15 per group) were used for cognitive testing on the touchscreen tasks as outlined previously^7^. Male mice were employed due to logistical limitations in not being able to test both male and female mice within the same apparatus, thereby avoiding potential confounds associated with prior observations of erratic behaviour displayed by male mice in apparatus where females had previously been tested. Mice were maintained on a restricted diet at or above 85% of their free-feeding body weight during behavioral testing. Water was available *ad libitum* throughout the experiment. All experimental protocols were approved by UK Home Office project licences and conducted in accordance with the United Kingdom Animals (Scientific Procedures) Act (1986).

### Cognitive testing of mice in the rodent touchscreen operant system

Testing was conducted in a touchscreen-based automated operant system as that described previously^7^ that consisted of an operant chamber (21.6 × 17.8 × 12.7 cm) made of clear Perspex walls and a stainless steel grid floor, housed within a sound- and light-attenuating box (40 × 34 × 42 cm) (Med Associates, St Albans, VT). A dispenser delivering reward pellets (14 mg, BioServ, Frenchtown NJ) into a magazine, a house light and a tone generator were located at one end of the chamber. At the opposite end of the chamber was a flat-screen monitor equipped with an infrared touchscreen (16 × 21.2 cm) (Craft Data Limited, Chesham, UK). The touchscreen was covered by a black Perspex ‘mask’ with windows positioned in front of the touchscreen allowing the presentation of stimuli to be spatially localized and prevented the mouse from accidentally triggering the touchscreen. Stimuli presented on the screen were controlled by custom software (“MouseCat,” L.M. Saksida; Carola Romberg) and responses made via nose-pokes at the stimuli were detected by the touchscreen and recorded by the software.

### Object-location paired-associates learning

Animals were pre-trained through several phases for instrumental operant conditioning as that previously described^7^. Once animals successfully completed the pre-training phases, mice were moved onto the task proper. In the object-location paired-associates learning test, mice were tested for the ability to associate between objects (shapes) and locations on the touchscreen^20^,^21^. There were three objects (flower, plane, and spider) and three correct spatial locations (left, centre, and right, respectively). For each trial, only 2 objects were presented; one object in its correct location (S+) and the other object in one of two incorrect locations (S−). There were six possible trial types^21^, so that the flower was rewarded only when presented in the left location, the plane was rewarded only when presented in the middle location, and the spider was rewarded only when presented in the right location. A nose-poke to the correct S+ resulted in delivery of a reward and incorrect responses resulted in a 5 s time-out period, followed by correction trial whereby the trial was repeated until the mouse made a correct choice. Nose-pokes to response windows in which no stimulus was presented were ignored. Mice were given 36 trials per session per day for 50 sessions to solve this complex problem-solving task. Mice were run in parallel in 20 sets of chambers, and so the experiment lasted approximately 60 days. Training on this task, which taps into more complex cognition, requires more training than some standard methods of testing rodent cognition (e.g., novel object recognition, fear conditioning or water maze) but is mitigated by many advantages^6^,^18^. For trial block analysis, data from 45 sessions (3 blocks of 15 sessions) was used. Group differences were analyzed using a mixed between-within subjects ANOVA with genotype as the between-subjects factor and block as the within-subjects factor. A paired samples t-test was used for *post hoc* analysis to assess significant between x within-subjects interaction effects. All values reported represent mean ± standard error of the mean.

### Human controls and DLG2 CNV participants

Control participants (males n = 14; females n = 16, ages 23–63, NART (National Adult Reading Test) score ≤120) with no history of major mental illness were recruited through the Family and Population Genetic Study of Mental Health at the Royal Edinburgh Hospital. Four individuals with CNV deletions within *DLG2* participated in the current study (see Table 1). As previously described^7^, initial discovery of 4 unrelated cases of *DLG2* CNV carriers (includes Participants 1 and 3) was made in the International Schizophrenia Consortium Genome Wide Association Study (GWAS)^11^ from 1115 Scottish schizophrenia cases (0.36%). From 978 Scottish control individuals screened, none was found to have this CNV. Expanding the pedigree of one of the individuals (Participant 1) discovered in the GWAS led to finding 2 more individuals within the same family with *DLG2* CNVs (2 daughters; 1 diagnosed with schizophrenia (Participant 2) and 1 not affected (Participant 4)). The study was carried out in accordance with the approved guidelines of the Multi-Centre Research Ethics Committee for Scotland and all patients or their legal proxy gave written informed consent for the collection of DNA samples for use in genetic studies.

### Human touchscreen testing using the rodent object-location paired associates learning test

The rodent touchscreen object-location paired associates learning test was adapted to be administered using a human touchscreen tablet. The test was run in a similar way to that described above for mice. For each trial, only 2 objects were presented; one object in its correct location and the other object in one of two incorrect locations. In order to make the testing method comparable to the rodent protocol, participants were given minimal instructions. Participants were instructed that objects would appear on the screen and they were required to make a response by touching an object and that the computer would inform them if their choice was correct or not. A correct response resulted in a ‘CORRECT’ message being displayed on the screen and incorrect responses resulted in an ‘INCORRECT – PLEASE TRY AGAIN’ message, followed by a correction trial. Participants were required to complete 72 trials. For trial block analysis, 3 blocks of 24 trials was used. Group differences were analyzed using the Friedman test and a Wilcoxon signed-rank test to assess *post hoc* differences. Direct comparison of mouse to human performance was analysed using a mixed between-within subjects ANOVA with genotype (controls/WT, *DLG2* CNV deletion/*Dlg2*^−/−^) and species (human, mouse) as the between-subjects factors and block of trials as the within-subjects factor. All values reported represent mean ± standard error of the mean.

## Additional Information

**How to cite this article**: Nithianantharajah, J. *et al.* Bridging the translational divide: identical cognitive touchscreen testing in mice and humans carrying mutations in a disease-relevant homologous gene. *Sci. Rep.* **5**, 14613; doi: 10.1038/srep14613 (2015).

## Supplementary Material

Supplementary Information

## Figures and Tables

**Figure 1:**
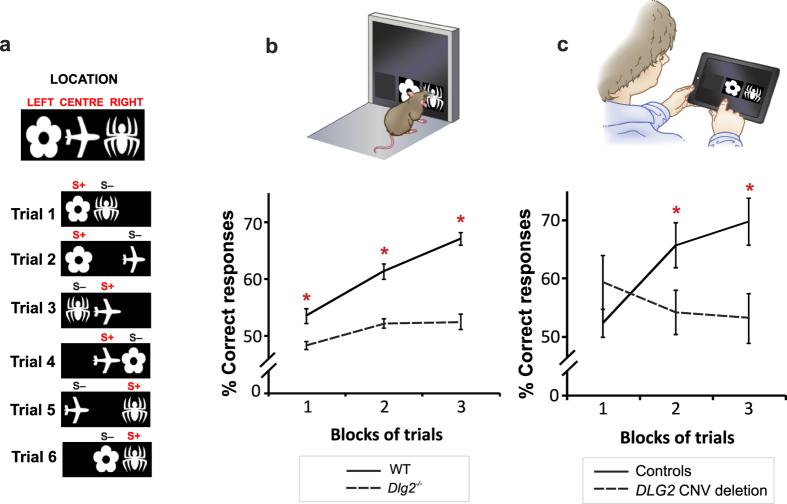
Mice and humans with mutations in *Dlg2* tested on the identical rodent touchscreen object-location paired associates task. (**a**) Rodent object-location paired associates learning test with 6 trials types (S+, correct; S−, incorrect). (**b,c**) Performance across blocks of training trials for *Dlg2*^–/–^ mice (**b**) and individuals with *DLG2* CNV deletions (**c**). Mouse: significant differences in genotype, blocks of trials and genotype x block interaction (p < 0.005); *post hoc* analysis revealed *Dlg2*^*−/−*^mice were significantly impaired compared to WT mice across all 3 blocks of trials (p < 0.01). Human: controls show significant differences across blocks of trials (p < 0.001); *post hoc* analysis revealed improved performance from blocks 1 to 2 and 3 (p < 0.005). In contrast, *DLG2* participants were impaired and failed to show this progressive acquisition (p = 0.549). *p < 0.01.

**Table 1:** Details of *DLG2* CNV deletion participants and controls. Gender (F, female; M, male), Age, Diagnosis and IQ (National Adult Reading Test, NART) is indicated for each participant and controls (NART represented as mean ± SEM).

	Gender	Age	Diagnosis	IQ (NART score)
Participant 1	F	67	Schizophrenia	Normal (105)
Participant 2	F	28	Schizophrenia	Severe Learning Disability
Participant 3	F	61	Schizophrenia	Normal (105)
Participant 4	F	24	Nil	Normal (105)
Controls	F + M(n = 30)	23–63	Nil	Normal (109.8 ± 1.2)
