# Supplementary material for: Bridging the translational divide: identical cognitive touchscreen testing in mice and humans carrying mutations in a disease-relevant homologous gene
Source: Sci Rep. 2015 Oct 1;5:14613. doi: 10.1038/srep14613 (PMC4589696; doi:10.1038/srep14613)
Supplement: Supplementary Information [file srep14613-s1.pdf]

Supplemental Information

Bridging the translational divide: identical cognitive touchscreen testing in mice and humans carrying mutations in a disease-relevant homologous gene

Authors: J. Nithianantharajah[1,2#], A.G. McKechanie[3,4], T.J. Stewart[4], M. Johnstone[4], D.H. Blackwood[4], D. St Clair[5], S.G.N. Grant[1,2]*, T.J. Bussey[6]* & L.M. Saksida[6]*

[1]Genes to Cognition Programme, Centre for Clinical Brain Sciences & Centre for Neuroregeneration, University of Edinburgh, Chancellors Building, 47 Little France Crescent, Edinburgh UK EH16 4SB

[2]Genes to Cognition Programme, Wellcome Trust Sanger Institute, Hinxton Cambridgeshire UK CB10 1SA

[3]The Patrick Wild Centre, University of Edinburgh, Royal Edinburgh Hospital, Edinburgh UK EH10 5HF

[4]Division of Psychiatry, University of Edinburgh, Royal Edinburgh Hospital, Edinburgh UK EH10 5HF

[5]Institute of Medical Sciences, University of Aberdeen, Aberdeen UK AB25 2ZD

[6]Department of Psychology, University of Cambridge & The MRC and The Wellcome Trust Behavioral and Clinical Neuroscience Institute, University of Cambridge, Cambridge UK CB2 3EB

[#]Current address: Florey Institute of Neuroscience and Mental Health, University of Melbourne, Parkville Victoria Australia 3052

* These authors co-supervised the project

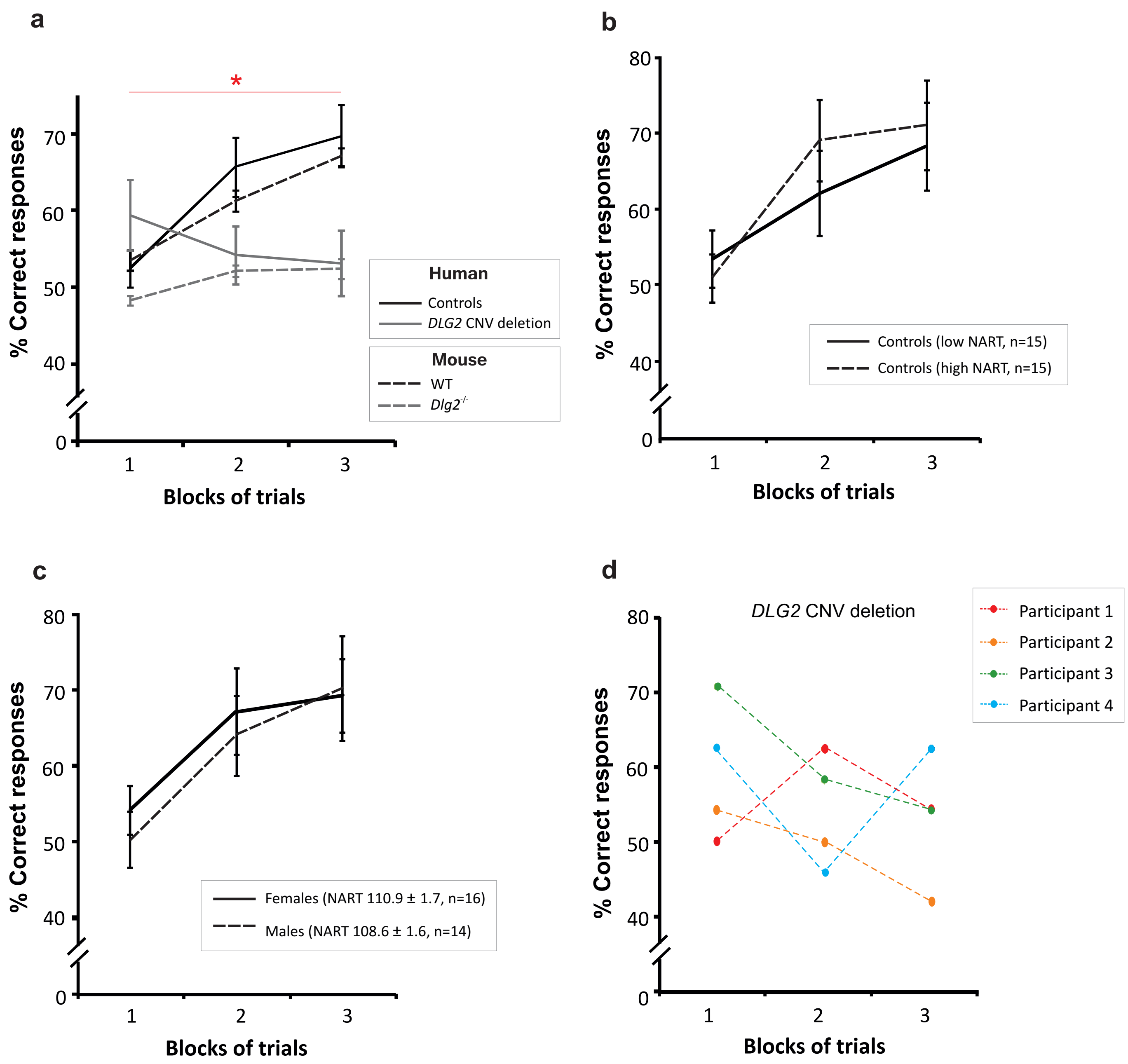

Supplementary Figure 1: Performance of controls and *DLG2* CNV deletion participants on the rodent touchscreen object-location paired associates task.

a. Direct comparison of mouse to human performance showed a significant genotype x block interaction (*p=0.005), but no differences between species (p=0.443).

b. Control group performance (percentage of correct responses) across blocks of trials based on median split of NART scores (low NART = 95-110; high NART = 110-120) showed no significant differences (p=0.69).

c. Control group performance based on gender also showed no differences (p=0.74).

d. Scatter plot of performance of individual *DLG2* CNV subjects indicates none of the 4 subjects showed the progressive acquisition and increase in performance across blocks of trials observed in the control group.
